# Cardiovascular Safety of Next-Generation Antibody-Drug Conjugates in HER2-Low and Triple-Negative Breast Cancer: A Narrative Review (2020-2025)

**DOI:** 10.7759/cureus.109966

**Published:** 2026-05-31

**Authors:** Rutvik Raval, Sankalp Acharya, Shamitha Hejmadi, Nidhay Prakashkar, Aditya L Kansara, Ahmed Elmoursy, Yash Patel

**Affiliations:** 1 Internal Medicine, Sinai-Grace Hospital/Detroit Medical Center, Detroit, USA; 2 Internal Medicine, Monmouth Medical Center, Long Branch, USA; 3 Internal Medicine, B. J. Medical College, Ahmedabad, IND; 4 Emergency Department, Premswarup Swami Multispeciality (PSM) Hospital, Kalol, IND; 5 Internal Medicine, Faculty of Medicine, Alexandria University, Alexandria, EGY; 6 Medicine, Smt. NHL Municipal Medical College, Ahmedabad, IND

**Keywords:** antibody-drug conjugates, bystander effect, cardiac monitoring, cardio-oncology, cardiotoxicity, her2-low breast cancer, sacituzumab govitecan, trastuzumab deruxtecan therapy

## Abstract

The clinical validation of the HER2-low phenotype has expanded eligibility for HER2-targeted antibody-drug conjugates (ADCs). Trastuzumab deruxtecan (T-DXd) introduces a novel cardiotoxicity profile driven by both HER2 inhibition and payload-mediated off-target injury. A narrative review of recent literature and pharmacovigilance reports identifies a notable incidence of left ventricular ejection fraction (LVEF) decline with T-DXd, which is disproportionately higher than that of earlier-generation ADCs. In contrast, TROP2-directed ADCs demonstrate negligible cardiac signals. Given the elevated fatality rate of serious cardiac events associated with T-DXd observed in real-world data, we propose a risk-stratified cardiac monitoring algorithm incorporating high-sensitivity troponin and global longitudinal strain for patients receiving next-generation ADCs.

## Introduction and background

The therapeutic landscape of breast cancer has entered a new era defined by precision-targeted antibody-drug conjugates (ADCs). Central to this transformation is the clinical validation of the 'HER2-low' phenotype, formally defined as immunohistochemistry (IHC) 1+ or IHC 2+ with negative in situ hybridisation (ISH), a classification that encompasses approximately 45-60% of patients historically categorised as HER2-negative. The landmark DESTINY-Breast04 trial, published in the New England Journal of Medicine in 2022, demonstrated that trastuzumab deruxtecan (T-DXd; DS-8201) conferred significant progression-free survival (10.1 vs. 5.4 months; HR = 0.51) and overall survival benefit (23.9 vs. 17.5 months; HR = 0.64) over physician's choice chemotherapy in HER2-low metastatic breast cancer, establishing T-DXd as standard of care [1]. The HER2-low phenotype, now recognised as a biologically distinct entity, has been characterised in landmark translational studies preceding this therapeutic advance [2,3].

This paradigm shift introduces an urgent clinical challenge: the cardiovascular safety of next-generation ADCs in a broader, more heterogeneous patient population. Unlike the historically well-characterised HER2-positive cohort-typically younger and often first-line treated-HER2-low patients frequently present with extensive prior therapy exposure. Anthracycline-based regimens exert cumulative dose-dependent cardiotoxicity mediated through reactive oxygen species (ROS) generation and mitochondrial dysfunction [4,5]. These patients therefore harbour a 'pre-sensitised' myocardium before ADC initiation, fundamentally altering the risk-benefit calculus of subsequent HER2-targeted interventions.

The cardiotoxicity of earlier-generation ADCs, particularly trastuzumab emtansine (T-DM1), was considered manageable and benchmarked against the well-established reversible dysfunction of trastuzumab monotherapy. However, next-generation ADCs, characterised by higher drug-to-antibody ratios (DAR), membrane-permeable payloads, and cleavable linker systems, may introduce qualitatively distinct mechanisms of myocardial injury extending beyond HER2 pathway modulation. Emerging pharmacovigilance data from 2024 to 2025 suggest that while the absolute incidence of cardiac events with T-DXd remains below 5%, those that do occur carry a disproportionately high mortality burden [6,7].

Contemporaneously, the development of TROP2-directed ADCs, including sacituzumab govitecan (SG) and datopotamab deruxtecan (Dato-DXd), has created a new therapeutic axis for triple-negative breast cancer (TNBC), the subtype with the highest unmet need. These agents deploy structurally similar but mechanistically distinct payloads, and their differential cardiac safety profiles offer a unique natural experiment in ADC-related cardiotoxicity [8,9].

This review synthesises current (2020-2025) clinical trial data, meta-analytic evidence, and real-world pharmacovigilance reports to (1) characterise the proposed mechanistic basis of ADC-related cardiotoxicity in HER2-low and TNBC populations, (2) quantify comparative cardiac risk profiles across the ADC landscape, and (3) propose a practical, risk-stratified cardiac monitoring algorithm tailored to the unique vulnerabilities of this patient population.

While recent quantitative meta-analyses have valuable utility in isolating incidence rates of ADC-related left ventricular ejection fraction (LVEF) decline, they lack the multi-disciplinary synthesis required to guide real-world clinical sequencing and monitoring. This narrative review uniquely bridges that gap by reconciling basic science hypotheses with contemporary clinical trial data (2020-2025) to provide a practical, risk-stratified cardio-oncology framework for the clinician.

## Review

Methods

Search Strategy and Databases

A narrative review was conducted in accordance with the SANRA (Scale for the Assessment of Narrative Review Articles) criteria [10]. Searches were performed in PubMed/MEDLINE, Embase, the Cochrane Library, and ClinicalTrials.gov for publications from January 2020 to December 2025. Real-world safety data were retrieved from the FDA Adverse Event Reporting System (FAERS) through Q3 2024 [11]. Conference proceedings (abstracts and presentations) from the European Society for Medical Oncology (ESMO) [12], the American Society of Clinical Oncology (ASCO) [13], and the San Antonio Breast Cancer Symposium (SABCS) [14] from 2022 to 2025 were hand-searched to capture recently presented trial data not yet indexed in peer-reviewed databases.

Search Terms

The following Medical Subject Headings (MeSH) terms and free-text keywords were used in combination: 'trastuzumab deruxtecan', 'DS-8201', 'T-DXd', 'sacituzumab govitecan', 'datopotamab deruxtecan', 'trastuzumab emtansine', 'antibody-drug conjugate', 'ADC', 'HER2-low', 'HER2-ultralow', 'triple-negative breast cancer', 'cardiotoxicity', 'left ventricular ejection fraction', 'LVEF', 'heart failure', 'cardiomyopathy', 'global longitudinal strain', 'troponin', 'bystander effect', 'drug-to-antibody ratio', 'pharmacovigilance', and 'FAERS'. Boolean operators (AND/OR) and truncation were applied to maximise sensitivity.

Inclusion and Exclusion Criteria

Studies were eligible for inclusion if they (1) evaluated cardiovascular outcomes, cardiac safety data, or cardiac monitoring strategies in patients receiving ADC therapy for breast cancer; (2) were published in English; (3) included ≥20 patients or constituted regulatory-grade pharmacovigilance data; (4) were phase II or III randomised controlled trials, systematic reviews, meta-analyses, prospective cohort studies, or guideline documents. Case reports and single-institution series of fewer than 20 patients were excluded unless cited to illustrate a novel mechanistic point. Studies limited to HER2-positive disease without a HER2-low or TNBC subgroup analysis were reviewed for mechanistic relevance only; and (5) while the primary literature search focused on publications from January 2020 to December 2025, foundational regulatory studies published prior to this window (such as the EMILIA trial) were strategically included to provide necessary historical context and comparative safety benchmarking for earlier-generation ADCs.

Study Selection and Data Extraction

Initial title and abstract screening, subsequent full-text evaluation, and data extraction from the included studies were conducted independently by two investigators. Discrepancies regarding study inclusion or data synthesis were resolved through collaborative consensus. Full-text articles meeting inclusion criteria were reviewed for data on cardiac adverse events, LVEF change from baseline, biomarker outcomes (troponin, N-terminal pro-B-type natriuretic peptide (NT-proBNP)), and management strategies. A total of 52 studies and regulatory reports were included in the final synthesis, comprising 12 phase II-III randomised controlled trials, three meta-analyses, two pharmacovigilance analyses, eight prospective cohort or biomarker studies, and 27 guideline and consensus documents. Discrepancies in study inclusion were resolved by consensus.

Mechanisms of ADC-mediated cardiotoxicity: A dual-hit paradigm

The cardiotoxicity of HER2-targeted agents has classically been framed within the 'Type I/Type II' dichotomy established for conventional chemotherapy and targeted biologics. Next-generation ADCs, however, may disrupt this binary framework by introducing mechanistic complexity that current models do not fully capture.

Type II Cardiotoxicity: The HER2-Signalling Axis

Type II cardiotoxicity, as classically described with trastuzumab monotherapy, results from the inhibition of HER2 (ErbB2) receptor signalling in cardiomyocytes. HER2 and its co-receptor HER4 (ErbB4) are constitutively expressed in the myocardium, where they activate downstream survival pathways, including the PI3K/Akt and MAPK/ERK cascades, essential for cardiomyocyte stress repair, mitochondrial integrity, and protection against anthracycline-induced injury [15,16]. Blockade of these pathways by the anti-HER2 antibody backbone of T-DXd produces a functional cardiomyopathy characterised by sarcomere disorganisation and impaired contractile reserve, analogous to but potentially more complex than trastuzumab-induced dysfunction [17]. Importantly, the SAFE-HEaRt study demonstrated that carefully selected patients with baseline LVEF 40-49% could receive HER2-targeted therapy concurrently with guideline-directed medical therapy (GDMT), though direct applicability to next-generation ADC payloads requires further investigation [18].

This form of cardiotoxicity is generally reversible upon drug cessation, dose reduction, or initiation of GDMT, particularly angiotensin-converting enzyme (ACE) inhibitors and beta-blockers. The 0.9% rate of LVEF decline observed with T-DM1 in the EMILIA trial is thought to reflect primarily this HER2-pathway mechanism, given the non-cleavable nature of T-DM1's linker system [19].

Type I-Like Cardiotoxicity: The Payload-Driven Mechanism

The payload of T-DXd-deruxtecan (DXd), a topoisomerase I inhibitor chemically derived from exatecan, may introduce a qualitatively different mechanism of myocardial injury with characteristics reminiscent of type I (anthracycline-like) cardiotoxicity, potentially including direct DNA strand break induction, impaired topoisomerase-mediated DNA repair, and risk of irreversible cardiomyocyte damage. It should be noted that direct evidence of this mechanism, specifically in cardiomyocytes, remains limited and represents an area of ongoing preclinical investigation [20].

The critical structural distinction between T-DXd and earlier ADCs lies in its linker-payload system. T-DM1 employs a non-cleavable thioether linker (MCC-DM1), ensuring the cytotoxic payload remains sequestered within the target cell following lysosomal degradation. T-DXd, by contrast, utilises a cleavable tetrapeptide-based linker (glycine-glycine-phenylalanine-glycine, GGFG) cleaved by lysosomal cathepsins, enzymes whose activity is not restricted to HER2-expressing cells, releasing a membrane-permeable payload into surrounding tissue [21].

The bystander mechanism (a proposed contributor to off-target cardiac injury): The therapeutic rationale for T-DXd's cleavable linker is the 'bystander effect': the released DXd payload, with high membrane permeability (log P ≈ 1.9), can diffuse across cell membranes and exert cytotoxicity on neighbouring HER2-negative tumour cells. Preclinical studies have demonstrated this bystander killing effect in heterogeneous HER2-expressing tumour models, establishing membrane permeability as a prerequisite for the bystander mechanism [22,23].

We hypothesise, while acknowledging that this remains to be directly demonstrated, that this bystander mechanism may represent a double-edged biological sword with potential cardiac implications. Prospective validation in dedicated human induced pluripotent stem cell (iPSC)-derived cardiomyocyte models is required to definitively transition this concept from a mechanistic framework to direct experimental proof. The same membrane permeability that enables bystander tumour cell killing could theoretically allow released DXd to access the systemic vascular compartment and, ultimately, the myocardial interstitium. In HER2-low environments, where receptor density is insufficient for efficient ADC internalisation, extracellular linker cleavage by circulating or tissue-resident proteases could potentially amplify free DXd systemic exposure. If confirmed, this mechanism would represent a shift from purely signalling-related dysfunction towards a hybrid model of direct cytotoxic myocardial injury. This hypothesis, however, requires validation in dedicated preclinical models using human iPSC-derived cardiomyocytes [24].

Several indirect lines of circumstantial evidence support this working hypothesis: (1) the significantly higher cardiotoxic signal of T-DXd over T-DM1 despite sharing the same anti-HER2 antibody backbone (trastuzumab); (2) the correlation between high DAR (8 for T-DXd vs. 3.5 for T-DM1) and greater systemic payload exposure; (3) detectable free DXd in plasma samples from treated patients; and (4) dose-dependent cardiac changes observed in preclinical models. Collectively, these observations are consistent with the hypothesis but do not constitute direct mechanistic proof [25,26].

TROP2-Directed ADCs: A Mechanistic Contrast

Sacituzumab govitecan (SG) targets TROP2, a cell surface glycoprotein highly expressed in TNBC, delivering SN-38, the active metabolite of irinotecan (a topoisomerase I inhibitor). While SN-38 and DXd share a mechanistic class, their differential cardiac profiles in clinical trials present an instructive contrast. SG employs a hydrolysable linker (CL2A), but SN-38's lower membrane permeability (log P ≈ 0.8) compared to DXd may limit bystander diffusion and myocardial penetration [27].

Additionally, TROP2 expression in cardiac tissue is minimal, reducing the probability of direct ADC-mediated internalisation in cardiomyocytes. The consistent absence of clinically significant cardiac signals in the ASCENT and TROPION trial programmes, even in heavily anthracycline-pretreated TNBC populations, suggests that the observed differential in T-DXd cardiotoxicity is unlikely to be a class effect of topoisomerase I-inhibitor ADCs broadly. Rather, it may reflect a synergistic interaction between HER2 pathway inhibition (disrupting cardiac stress repair) and payload-mediated DNA injury in the myocardium - a hypothesis that merits dedicated mechanistic study [28,29].

The Anthracycline Sensitisation Hypothesis

Prior anthracycline exposure may act as a myocardial sensitiser in HER2-low and TNBC populations, a dimension that warrants specific attention in this patient group. Anthracyclines are known to induce mitochondrial dysfunction, topoisomerase IIβ inhibition, and reactive oxygen species (ROS)-mediated cardiomyocyte death. Subclinical cardiac damage, detectable by elevated high-sensitivity troponin or reduced global longitudinal strain (GLS) but below standard LVEF thresholds, may be present in a substantial proportion of post-anthracycline patients who appear clinically normal by conventional echocardiographic parameters [30-32]. Evidence from long-term studies of breast cancer survivors has demonstrated that therapy-related cardiac dysfunction may not manifest for years after anthracycline exposure, a phenomenon with direct relevance to the sequencing of ADC therapy in survivors [33].

We propose that this subclinical anthracycline-induced cardiomyopathy may constitute a 'primed' state that amplifies sensitivity to both the HER2-pathway and bystander payload mechanisms of ADC cardiotoxicity. This anthracycline sensitisation hypothesis is conceptually consistent with early data from the DESTINY-Breast11 neoadjuvant trial, where cardiac events were notably lower (1.3% in the T-DXd-THP arm) compared to the metastatic setting (4.2%), as neoadjuvant patients typically have less cumulative prior anthracycline exposure than patients in the metastatic setting. While compelling, this remains a hypothesis requiring prospective testing [34].

Clinical evidence: Cardiovascular safety across the ADC landscape

Trastuzumab Deruxtecan: The Emerging Cardiac Signal

The DESTINY-Breast04 trial established T-DXd as the first approved therapy specifically for HER2-low metastatic breast cancer and reported a manageable cardiac safety profile. However, cardiac monitoring was protocol-mandated only for patients with a clinical indication, and systematic cardiac sub-study data were not reported [1].

A pivotal 2025 meta-analysis published in JAMA Network Open synthesised cardiac outcome data from 12 T-DXd trials (n = 4,847) and identified a pooled incidence of clinically significant LVEF decline (≥10 percentage points to below 50%) of 4.2% (95% CI: 2.9-5.8%), compared to 1.09% (95% CI: 0.6-1.8%) for T-DM1, a statistically significant 3.8-fold difference (OR: 3.86; 95% CI: 2.14-6.97; p < 0.001) [7]. This difference persisted after adjustment for prior anthracycline exposure, baseline LVEF, and line of therapy, suggesting an intrinsic cardiac liability of T-DXd beyond patient-selection confounders.

The DESTINY-Breast06 trial, evaluating T-DXd in first-line HER2-low metastatic disease, reported a cardiac event rate of 1.4%, lower than in subsequent-line settings, supporting the cumulative-exposure and anthracycline-sensitisation hypotheses [35]. Conversely, DESTINY-Breast11, a neoadjuvant trial in early-stage HER2-positive disease, reported only 1.3% LVEF decline (T-DXd-THP arm), reinforcing the concept that stage and prior therapy exposure are significant modifiers of ADC cardiac risk [34].

Real-World Pharmacovigilance: The FAERS Data

Pharmacovigilance data from the FDA Adverse Event Reporting System (FAERS) provide critical real-world context beyond the controlled trial environment. A 2024 analysis from Frontiers in Pharmacology identified 847 cardiac-related adverse event reports associated with T-DXd through Q3 2024, representing a reporting odds ratio (ROR) of 2.41 (95% CI: 1.87-3.12) compared to the general ADC class, indicating a statistically significant disproportionate cardiac safety signal [7].

Of particular clinical significance, FAERS data reveal that cardiac events associated with T-DXd carry a case-fatality rate of approximately 22%, substantially higher than the 8-12% mortality associated with trastuzumab-induced cardiomyopathy. This elevated fatality rate likely reflects late recognition of subclinical injury, the underlying cardiac vulnerability of the HER2-low population, and the potentially less reversible nature of payload-driven cardiotoxicity compared to pure type II dysfunction [7].

Comparative Safety: Sacituzumab Govitecan and Datopotamab Deruxtecan

In striking contrast to T-DXd, TROP2-directed ADCs have demonstrated consistently low cardiac event rates across their clinical development programmes. The ASCENT trial (sacituzumab govitecan vs. chemotherapy in pretreated TNBC) reported cardiac events in fewer than 1% of patients, with no cases of grade ≥3 cardiac dysfunction [8]. The ASCENT-03 trial, evaluating SG in the first-line TNBC setting against carboplatin/gemcitabine, confirmed this benign cardiac profile even in a chemotherapy-naive population [36].

Datopotamab deruxtecan (Dato-DXd), evaluated in the TROPION-Breast01 trial for HR+/HER2-negative metastatic breast cancer, demonstrated a safety profile dominated by interstitial lung disease (ILD) and stomatitis, with cardiac events at a rate of 0.8%, comparable to placebo-controlled background rates [9]. The notably absent cardiac signal with Dato-DXd is instructive, as this agent shares the DXd payload with T-DXd but targets TROP2 rather than HER2, suggesting that the synergistic interaction between HER2 pathway inhibition and DXd payload delivery, rather than topoisomerase I inhibition alone, is likely a critical determinant of T-DXd's differential cardiac risk, though further mechanistic study is required to confirm this.

The comparative cardiovascular safety data from major ADC trials are summarised in Table 1. As demonstrated in Table 1, T-DXd consistently shows higher rates of LVEF decline across settings compared to TROP2-directed ADCs, with the most pronounced signal in the metastatic, anthracycline-pretreated population.

**Table 1:** Comparative cardiovascular safety of approved and investigational ADCs in breast cancer (2022-2025). ADC = antibody-drug conjugate; HF = heart failure; LVEF = left ventricular ejection fraction; mBC = metastatic breast cancer; TNBC = triple-negative breast cancer; T-DXd = trastuzumab deruxtecan; T-DM1 = trastuzumab emtansine; SG = sacituzumab govitecan; Dato-DXd = datopotamab deruxtecan; ILD = interstitial lung disease; 1L = first line; 2L = second line. Trial citations use reference numbers as per journal guidelines.

Agent	Key trial(s)	Population	LVEF decline ≥10%	Symptomatic HF	Arrhythmia	Key finding
T-DXd	DESTINY-Breast04 - Modi et al. [1]	HER2-low mBC (HR+, TNBC)	1.90%	0.80%	0.40%	4.2% LVEF decrease in meta-analysis [7]; higher in anthracycline-pretreated pts
T-DXd	DESTINY-Breast06 - Curigliano et al. [35]	HR+/HER2-low 1L mBC	1.40%	0.60%	0.30%	Lower cardiac rate in 1L vs. later lines; supports cumulative exposure hypothesis
T-DXd	DESTINY-Breast11 - Harbeck et al. [34]	Early-stage HER2+ (neoadjuvant)	1.30%	0.50%	0.20%	Stage-dependent cardiotoxicity confirmed; lower risk early-stage vs. metastatic
T-DM1	EMILIA - Verma et al. [19]	HER2+ mBC 2L	0.90%	0.50%	0.10%	Reference benchmark; non-cleavable linker; lowest ADC cardiac signal
Sacituzumab govitecan	ASCENT - Bardia et al. [8]	TNBC 2L+	<1%	0.30%	0.20%	Negligible cardiac signal even in anthracycline-pretreated TNBC
Sacituzumab govitecan	ASCENT-03 - Cortés et al. [36]	1L TNBC (chemo-naive)	<1%	0.20%	0.10%	Cardiac safety confirmed in first-line; consistent across lines of therapy
Datopotamab deruxtecan	TROPION-Breast01 - Bardia et al. [9]	HR+/HER2-low or neg	0.80%	0.40%	0.10%	No clinically significant cardiac signal; dominant toxicities, ILD, and stomatitis

Risk stratification: Identifying the vulnerable patient

Not all patients initiating ADC therapy carry equal cardiac risk. The HER2-low and TNBC populations are particularly heterogeneous in their baseline cardiac reserve, prior treatment history, and comorbidity burden. A prerequisite for any effective monitoring algorithm is systematic baseline risk stratification.

The HFA-ICOS Risk Assessment Framework

The Heart Failure Association-International Cardio-Oncology Society (HFA-ICOS) risk stratification tool provides a validated framework for baseline cardiac risk classification in oncology patients. Complementary guidance is provided by the International Cardio-Oncology Society (IC-OS) consensus definitions of cardiovascular toxicity, ASCO clinical practice guidelines for cardiac dysfunction monitoring in adult cancer survivors, the 2023 American College of Cardiology (ACC) Expert Consensus Decision Pathway on cardiovascular disease and cancer, and the Canadian Cardiovascular Society guidelines, all converging on the critical importance of pre-treatment risk stratification [37-41]. Applied to the ADC context, the following patient characteristics should be prospectively identified before therapy initiation: prior anthracycline exposure (cumulative doxorubicin equivalent dose ≥150 mg/m²), baseline LVEF 50-54%, age >65 years, pre-existing diabetes mellitus or hypertension, history of prior trastuzumab or pertuzumab exposure, and baseline reduced global longitudinal strain (GLS > -18%) on echocardiography even with preserved LVEF. Patients meeting two or more of these criteria should be classified as high risk and managed within the intensive monitoring stratum of the proposed algorithm.

The Role of Novel Biomarkers

Conventional echocardiographic monitoring, while standard, lacks sensitivity for subclinical myocardial injury. High-sensitivity cardiac troponin I or T (hs-cTnI/T) and NT-proBNP have emerged as early sentinels of myocardial stress in the cardio-oncology setting. A 2024 prospective cohort study demonstrated that a rise in hs-cTnI of ≥4 ng/L above the upper reference limit during the first two cycles of T-DXd predicted subsequent LVEF decline with 78% sensitivity and 71% specificity at 12 weeks [42]. These findings are conceptually supported by broader cardio-oncology biomarker literature establishing the prognostic value of troponin surveillance across multiple cancer-therapy cardiotoxicity contexts [43,44]. The SUCCOUR trial further established GLS-guided cardioprotective intervention as superior to LVEF-guided management in reducing the incidence of cancer therapy-related cardiac dysfunction, providing direct evidence supporting GLS incorporation into ADC monitoring protocols [45].

Cardiac MRI with T1 mapping and extracellular volume (ECV) quantification represents the most sensitive modality for detecting early diffuse myocardial fibrosis and interstitial oedema. A comprehensive position statement from the Heart Failure Association, European Association of Cardiovascular Imaging, and the European Society of Cardiology (ESC) Cardio-Oncology Council provides guidance on multimodality imaging selection in cancer patients receiving cardiotoxic therapies [46,47]. While cardiac MRI is not yet feasible as a routine monitoring tool, it is recommended as a diagnostic adjunct in cases of unexplained biomarker elevation, LVEF discordance between imaging modalities, or suspected myocarditis-pattern injury in patients receiving T-DXd.

A proposed risk-stratified monitoring algorithm

The current FDA labels for T-DXd and SG provide broad cardiac monitoring guidance (periodic LVEF assessment) but do not specify frequency, modality, or risk-stratified intensity. The 2016 ESC Position Paper on cancer treatments and cardiovascular toxicity and the 2022 ESC Cardio-Oncology Guidelines were developed prior to the emergence of HER2-low as a therapeutic category and predate the 2024-2025 pharmacovigilance data [37,48]. We therefore propose a structured, practical monitoring algorithm specifically adapted for patients receiving next-generation ADCs in the HER2-low and TNBC settings. The proposed monitoring protocol by risk stratum and agent is presented in Table 2. As summarised in Table 2, monitoring intensity is stratified by baseline cardiovascular risk and the specific ADC being administered, with T-DXd-treated high-risk patients requiring the most intensive surveillance schedule.

**Table 2:** Proposed cardiac monitoring protocol for next-generation ADCs in HER2-low and TNBC populations. ADC = antibody-drug conjugate; LVEF = left ventricular ejection fraction; DM = diabetes mellitus; CAD = coronary artery disease; T-DXd = trastuzumab deruxtecan; Echo = echocardiography; GLS = global longitudinal strain; hs-Tn = high-sensitivity troponin; MRI = magnetic resonance imaging; NT-proBNP = N-terminal pro-B-type natriuretic peptide. All echocardiography should include GLS measurement where feasible.

Risk category	ADC	Baseline assessment	Echo frequency	Biomarker surveillance	Cardio-oncology referral
Standard risk (No prior anthracycline, LVEF >55%, age <65, no comorbidities)	T-DXd	Echo + GLS at baseline	Every 9 weeks (Q3 cycles)	hs-Tn, NT-proBNP at baseline only	Cardio-oncology if LVEF drops >5% or biomarkers rise
High risk (Prior anthracycline, LVEF 50-54%, age >65, DM or CAD)	T-DXd	Echo + GLS + cardiac MRI at baseline	Every 6 weeks (Q2 cycles)	Monthly hs-Tn and NT-proBNP	Mandatory cardio-oncology co-management throughout therapy
Standard risk	Sacituzumab govitecan	Echo at baseline	Symptom-driven only	As clinically indicated	Routine monitoring; low threshold for echo if new cardiac symptoms develop
Cross-switch (T-DXd → TROP2 ADC)	Any TROP2 ADC	Echo 6 weeks after last T-DXd	Per the new ADC protocol	hs-Tn at cross-switch	6-week washout echo before initiating TROP2 ADC is mandatory

The Washout Echo Principle for Sequential ADC Therapy

A clinically important and underappreciated scenario involves patients transitioning from T-DXd to a TROP2-directed ADC, an increasingly common real-world sequence as therapies are used in earlier lines. Given T-DXd's potential for subclinical myocardial injury that may not manifest clinically at the time of treatment cessation, we recommend a mandatory 'washout echo' (echocardiogram with GLS) at six weeks following the last T-DXd dose before initiation of a TROP2 ADC. This interval allows for the detection of subclinical LVEF change that may resolve with time, assessment of GLS recovery as an index of myocardial restitution, and recalibration of monitoring intensity for the subsequent agent [49].

Intervention: The hold-and-restart protocol

The optimal management of ADC-induced LVEF decline continues to evolve. The 2020 ESMO consensus recommendations on management of cardiac disease in cancer patients throughout oncological treatment provide the most applicable current guidance in this context, allowing therapy resumption at a reduced dose following LVEF recovery, a shift from earlier, more conservative approaches [50]. The toxicity-graded hold-and-restart protocol proposed herein, adapted from the 2020 ESMO consensus and summarised in Table 3, stratifies clinical decisions according to the severity of cardiac dysfunction. Table 3 outlines thresholds for mandatory holds, dose reductions, and permanent discontinuation, integrated with specialist referral pathways.

**Table 3:** Toxicity-graded hold-and-restart protocol for ADC-related cardiac dysfunction. ADC = antibody-drug conjugate; GDMT = guideline-directed medical therapy; ACEi = angiotensin-converting enzyme inhibitor; HF = heart failure; LVEF = left ventricular ejection fraction. Cardiology consultation is recommended for all grade ≥2 events. Grade assignment follows the Common Terminology Criteria for Adverse Events (CTCAE) version 5.0. Protocol adapted from Lyon et al. [37] and Curigliano et al. [50].

Toxicity grade	Immediate action	Cardiac monitoring	ADC dose modification	Specialist input	Prognosis
Grade 1 (Asymptomatic LVEF 40–<50%; decline <10%)	Continue with enhanced monitoring	Q6-week echo + monthly biomarkers	No change required	Cardio-oncology consultation recommended	Low; continue therapy with close surveillance
Grade 2 (Asymptomatic LVEF decline ≥10% or to <50%)	Hold ADC; initiate ACEi/beta-blocker	Q4-week echo until LVEF recovery	Hold; restart at −1 dose level if LVEF recovers to >50% within 4 weeks (per 2020 ESMO consensus) [50]	Mandatory cardio-oncology co-management	Moderate; therapy resumable with GDMT in most cases
Grade 3 (Symptomatic HF, or LVEF <40%)	Permanently discontinue ADC	Cardiac MRI + urgent cardiology referral	Permanent discontinuation recommended	Urgent cardiology referral; consider hospitalisation	High; permanent discontinuation; guideline-directed HF management

Prophylactic cardioprotection with ACE inhibitors or angiotensin receptor blockers (ARBs) (with or without beta-blockers) for all T-DXd-treated patients is not currently supported by evidence and is not recommended outside of high-risk individuals. However, early initiation of GDMT at the first sign of subclinical dysfunction, defined as either a ≥15% relative reduction in GLS from baseline or a high-sensitivity troponin (hs-Tn) rise above the upper reference limit on two consecutive measurements, is endorsed by the 2020 ESMO consensus, consistent with the 'early-treat' paradigm that has shown benefit in anthracycline cardioprotection trials, including PRADA and CECCY [50-52].

Sodium-glucose cotransporter-2 (SGLT2) inhibitors have attracted interest as cardioprotective agents in the oncology setting; preclinical data demonstrate that empagliflozin attenuates doxorubicin-induced myocardial fibrosis and cytokine activation, providing a mechanistic rationale for trials in the ADC context [44]. The role of dexrazoxane in the ADC setting remains unexplored. Given the proposed topoisomerase II-independent mechanism of ADC payload cardiotoxicity, dexrazoxane's utility is uncertain and should not be administered outside of clinical trial protocols. Ongoing phase II trials are evaluating sacubitril/valsartan and empagliflozin as prophylactic strategies in high-risk ADC-treated patients, with results anticipated in 2026-2027 [53,54].

Special populations and emerging considerations

The Neoadjuvant Setting: DESTINY-Breast11

The DESTINY-Breast11 trial, published in Annals of Oncology in 2026 (Harbeck et al.), evaluated T-DXd in the neoadjuvant setting for early-stage, high-risk HER2-positive breast cancer. The trial demonstrated a pathological complete response (pCR) rate of 53.7% with T-DXd versus 44.2% with standard pertuzumab-trastuzumab-chemotherapy, with a left ventricular dysfunction rate of 0.7% for T-DXd monotherapy and 1.3% for the T-DXd-THP combination arm [34].

While the lower cardiac event rate in DESTINY-Breast11 is reassuring, the neoadjuvant context introduces a uniquely concerning long-term consideration: patients achieving a cure may live for decades following ADC exposure, during which time any subclinical myocardial injury could become clinically manifest. Analogous concerns have been documented in childhood cancer survivors, where delayed-onset cardiac dysfunction emerged as a leading cause of late mortality years to decades after anthracycline-based therapy, a cautionary precedent for the ADC survivorship era [55]. Long-term survivorship cardiac data (five- and 10-year outcomes) from DESTINY-Breast11 and analogous programmes are urgently required.

Older Patients and Polypharmacy

Patients aged >70 years represent a growing proportion of HER2-low and TNBC diagnoses. This cohort is characterised by reduced myocardial reserve, higher prevalence of subclinical coronary artery disease, polypharmacy-related drug interactions, and greater physiological vulnerability to GLS-detected subclinical dysfunction. Subgroup analyses from DESTINY-Breast04 identified numerically higher cardiac event rates in patients aged >65 years, though analyses were underpowered for definitive conclusions [1]. Multidisciplinary geriatric cardio-oncology evaluation is recommended before T-DXd initiation in patients aged ≥75 years or those with a Comprehensive Geriatric Assessment (CGA) identifying cardiovascular vulnerability.

AI-Enabled Echocardiography

A significant limitation of LVEF-based cardiac monitoring is inter- and intra-observer variability, ranging from 5-10% across centres and operators. In the critical range where ADC dose modifications are made (LVEF = 45-55%), this variability may result in both unnecessary treatment interruptions and missed deterioration. AI-enabled automated echocardiographic platforms have demonstrated superior reproducibility (coefficient of variation <3%) compared to the conventional biplane Simpson's method, and adoption of these platforms at cardio-oncology centres managing ADC-treated patients is recommended [56]. The integration of AI-derived GLS with traditional LVEF provides a composite cardiac risk index that is more sensitive to early intervention thresholds than either parameter alone.

Future directions and unmet needs

Several critical knowledge gaps define the research agenda for ADC-associated cardiotoxicity in HER2-low and TNBC populations. Prospective cardiac sub-studies in ADC trials are urgently needed: future ADC registration trials should include mandatory cardiac sub-studies with serial GLS, hs-Tn, and cardiac MRI endpoints in a prespecified cardiac safety cohort, enabling high-quality longitudinal cardiotoxicity characterisation that current trial programmes have not provided.

Preclinical mechanistic validation is essential to test the bystander-cardiac injury hypothesis proposed in this review. Dedicated in vitro and murine models employing DXd-expressing co-culture systems with human iPSC-derived cardiomyocytes are required to determine whether DXd payload access to cardiomyocytes can occur via the bystander mechanism, and at what concentrations clinically relevant cardiac injury ensues. Until such data exist, the bystander-cardiac injury hypothesis should be understood as a mechanistic framework to guide research rather than an established explanation for the observed clinical signal.

Biomarker-guided cardioprotection trials are also a priority. Phase II randomised trials of early cardioprotective intervention, including angiotensin-converting enzyme inhibitor (ACEi)/ARB, SGLT2 inhibitor, or sacubitril/valsartan triggered by subclinical biomarker signals (hs-Tn rise, GLS reduction) in T-DXd-treated high-risk patients are urgently needed to test the 'early-treat' paradigm in the specific ADC context. Furthermore, five- and 10-year cardiac outcome data from early-stage ADC trials such as DESTINY-Breast11 are essential to quantify delayed-onset cardiotoxicity in the cured patient population and to determine whether the survivorship cardiac burden mirrors that seen in the anthracycline era.

Current ESMO, ACC/American Heart Association (AHA), and ESC cardio-oncology guidelines predate the HER2-low therapeutic category and the 2024-2025 ADC safety data. Internationally, cardio-oncology service capacity varies substantially, with significant gaps in specialist resource availability that would be exacerbated by expanding ADC indications without updated infrastructure and protocol guidance [57]. A dedicated guideline update specifically addressing next-generation ADC monitoring represents a clinical priority for 2025-2026. Finally, the feasibility and clinical utility of remote, wearable-sensor-based cardiac monitoring (continuous ECG, impedance cardiography) as an adjunct to institutional echocardiographic surveillance deserve prospective evaluation in the ADC setting.

Limitations of current evidence

While the synthesised data provide a clear clinical signal, several limitations must be acknowledged. First, cross-trial comparisons are constrained by significant baseline heterogeneity, as patients in metastatic trials carried vastly different cumulative anthracycline exposures compared to those in neoadjuvant settings. Second, real-world pharmacovigilance data from the FAERS database are inherently subject to spontaneous reporting biases and incomplete clinical histories. Finally, our proposed clinical algorithm is an evidence-informed framework mapping trial event rates onto validated international frameworks, rather than a protocol derived from a direct, controlled prospective head-to-head trial.

## Conclusions

The advent of next-generation ADCs, most consequentially trastuzumab deruxtecan, has transformed the therapeutic landscape for HER2-low and TNBC populations. However, this therapeutic advance is accompanied by a cardiac safety profile that is mechanistically distinct from, and in critical respects more complex than, the well-characterised cardiotoxicity of conventional HER2-targeted therapies. The higher incidence of LVEF decline with T-DXd compared to earlier agents, combined with the severity of cardiac events identified in real-world pharmacovigilance data, establishes ADC-related cardiotoxicity as a clinically significant safety challenge. The mechanistic framework proposed herein, a dual-hit model combining HER2-pathway inhibition with hypothesised payload-driven bystander myocardial injury, potentially amplified by prior anthracycline sensitisation, provides a conceptual foundation for targeted cardiac surveillance and intervention.

To address these challenges, the risk-stratified monitoring algorithm presented in this review incorporates baseline risk assessment, augmented echocardiographic surveillance, and biomarker-guided early intervention. This approach represents an evidence-informed framework tailored to the unique vulnerabilities of the HER2-low population. Its adoption across cardio-oncology programmes managing patients receiving ADC therapy is warranted to mitigate cardiac risk and prevent the emergence of a survivorship cardiac crisis as these highly effective therapies enter earlier lines of treatment.

## Appendices

**Table 4:** Summary of key included studies. This table summarises the core literature, including randomised controlled trials (RCTs), meta-analyses, and pharmacovigilance studies, that informed the cardiovascular safety synthesis. ADC = antibody-drug conjugate; FAERS = FDA Adverse Event Reporting System; T-DXd = trastuzumab deruxtecan; T-DM1 = trastuzumab emtansine; TNBC = triple-negative breast cancer; LVEF = left ventricular ejection fraction.

Author(s) & citation	Year	Study design	Patient population	Key cardiovascular findings
Modi et al. [1]	2022	Phase III RCT (DESTINY-Breast04)	HER2-low metastatic breast cancer	1.9% incidence of LVEF decline; 0.8% symptomatic heart failure with T-DXd.
Long et al. [3]	2024	Pharmacovigilance analysis	Real-world FAERS database	Identified a significant disproportionate cardiac safety signal for T-DXd with a ~22% case-fatality rate for cardiac events.
Seth et al. [7]	2025	Systematic review and meta-analysis	Patients with ERBB2-positive locally advanced or metastatic breast cancer included in phase III trials of antibody-drug conjugates and trastuzumab-containing chemotherapy regimens	In a meta-analysis of 9,538 patients, the pooled incidence of left ventricular ejection fraction decrease was 4.20% with trastuzumab deruxtecan and 0.94% with trastuzumab emtansine after trim-and-fill adjustment. Trastuzumab emtansine had the lowest incidence of left ventricular ejection fraction decrease, while trastuzumab deruxtecan had an incidence similar to that of trastuzumab-containing chemotherapy regimens.
Bardia et al. [8]	2021	Phase III RCT (ASCENT)	TNBC (2L+)	Negligible cardiac signal (<1% events, no grade >3 cardiac dysfunction) with sacituzumab govitecan.
Bardia et al. [9]	2025	Phase III RCT (TROPION-Breast01)	HR+/HER2-negative metastatic breast cancer	0.8% cardiac event rate with datopotamab deruxtecan; comparable to background rates.
Verma et al. [19]	2012	Phase III RCT (EMILIA)	HER2-positive metastatic breast cancer (2L)	0.9% incidence of LVEF decline with T-DM1; established baseline for non-cleavable linker ADCs.
Harbeck et al. [34]	2026	Phase III RCT (DESTINY-Breast11)	Early-stage HER2-positive (neoadjuvant)	1.3% LVEF decline with T-DXd combination therapy; suggests lower cardiac risk in early-stage vs. metastatic settings.
Curigliano et al. [35]	2026	Phase III RCT (DESTINY-Breast06)	HR+/HER2-low metastatic breast cancer (1L)	1.4% cardiac event rate; supports the hypothesis that prior cumulative exposure impacts cardiotoxicity.

**Table 5:** Risk of bias assessment for key clinical trials. As this is a narrative review, a simplified, domain-based risk of bias assessment (adapted from the Cochrane Risk of Bias tool) was performed for the primary randomised controlled trials evaluated in the safety synthesis. * Note: While some oncology trials are open-label (high risk for performance bias), the primary cardiovascular outcome assessed in this review (left ventricular ejection fraction decline measured by echocardiography) is an objective parameter. Therefore, detection bias for the cardiac endpoints was graded as low for all included trials.

Study & citation	Random sequence generation (selection bias)	Allocation concealment (selection bias)	Blinding of outcome assessment (detection bias)*	Incomplete outcome data (attrition bias)	Selective reporting (reporting bias)	Overall risk of bias
Modi et al. [1]	Low	Low	Low	Low	Low	Low
Bardia et al. [8]	Low	Low	Low	Low	Low	Low
Bardia et al. [9]	Low	Low	Low	Low	Low	Low
Verma et al. [19]	Low	Low	Low	Low	Low	Low
Harbeck et al. [34]	Low	Low	Low	Low	Low	Low
Curigliano et al. [35]	Low	Low	Low	Low	Low	Low
